# Characterization of Poly(vinyl alcohol)/Carboxymethyl Cellulose Blends with NaNO_2_ to Develop Active Packaging for Use with Meat

**DOI:** 10.1021/acsomega.5c06803

**Published:** 2025-11-24

**Authors:** Edivânia R. Lopes, Romicy D. Souza, Jéssica S. de Oliveira, Marcela C. de Jesus, Eduardo M. Ramos, Cristiane P. de Oliveira

**Affiliations:** † Departament of Rural and Animal Technology, State University of Southwest Bahia, Praça Primavera no 40, Bairro Primavera, Itapetinga 45700-000, Bahia, Brazil; ‡ Department of Food Science, Laboratory of Food Microbiology, Federal University of Lavras—UFLA, CEP, Lavras, Minas Gerais 37200-000, CP 3037, Brazil

## Abstract

Active packaging is an innovative solution to preserve sensory characteristics and prolong the shelf life of meat products. In this study, films based on mixtures of poly­(vinyl alcohol) (PVA) and carboxymethylcellulose (CMC) were prepared and incorporated with sodium nitrite at concentrations of 0, 50, 100, and 500 ppm. The films were characterized in terms of their physical, optical, mechanical, and thermal properties, and evaluated for their active performance in pork. The incorporation of nitrite did not affect the physical characteristics of the films, which had an average thickness of 0.12 mm, good transparency, and total solubility in water. Water vapor permeability (WVP) ranged from 3.20 × 10^–5^ to 4.05 × 10^–5^ *P*(g/m·d·Pa). The films demonstrated good strength and flexibility, with an average elongation of 179% and a modulus of elasticity between 38 and 44 MPa. FTIR analysis did not reveal any bands attributable to nitrite. In storage tests with pork, nitrite migration to the product was observed; however, there was no consistent inhibition of lipid oxidation, with TBARS values ranging from 0.57 to 2.54 mg MDA/kg. Taken together, the results indicate that PVA/CMC blends have potential as active films for meat applications, offering good mechanical properties and nitrite release capacity. However, optimizations are needed to improve the moisture barrier and antioxidant efficacy in the product.

## Graphical abstract

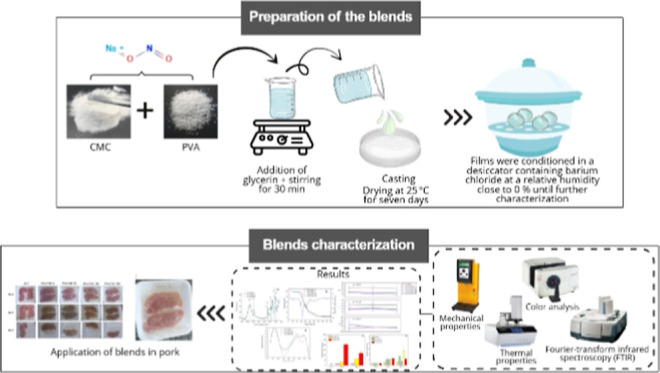

## 1 Introduction

An active film is defined as a package containing one or more active substances that interact with the environment around the food or with the food itself to provide specific benefits, such as delaying oxidation, inhibiting the growth of microorganisms, or absorbing unwanted gases.^1^ Among the components of interest for use in active packaging is sodium nitrite (NaNO_2_), a compound that has already been used in active coatings for meat applications.^2,3^ Studying polymer bases in which this additive can be properly incorporated for application in products such as meat and meat products is of great industrial interest.

NaNO_2_ is a widely used additive in meat products, as it performs crucial functions that make it irreplaceable. NaNO_2_ is responsible for forming the characteristic color of cured products by reacting with myoglobin to form nitrosomyoglobin, as well as having an antimicrobial action, acting mainly against *Clostridium botulinum*, and an antioxidant action, slowing down the oxidation of lipids and the formation of undesirable flavors and odors.^4,5^ Despite the importance of NaNO_2_ in the meat industry, there is concern about its cancerogenic potential since it is associated with the formation of nitrosamines, chemical compounds that increase the risk of certain types of cancer.^6^ Based on this problem, some studies have investigated different ways to reduce or replace the use of nitrite.^7–9^ However, the consumption of NaNO_2_ is only harmful if consumed above 150 ppm, as determined by legislation,^10–12^ and is entirely safe when consumed within limits.

One of the viable technological alternatives to reduce the adverse effects caused by NaNO_2_ is its incorporation into active packaging, where, through the controlled release of NaNO_2_, it is possible to reduce its concentration in the food and maintain its functional properties.^13^ In addition to the benefits of active packaging, the use of active materials produced with biodegradable compounds has been studied, given their environmental benefits and ability to preserve food.^14^

In a previous study, our research group elucidated the incorporation of nitrite into PVA films.^3^ In this study, it was possible to prove a reduction in lipid oxidation in pork, and we observed that improvements in the structure of the polymer could increase its physical characteristics and diversify its active function in the meat sector. One of the possibilities for improving film characteristics is to combine polymers to form blends, so we opted to use a mixture of poly­(vinyl alcohol) (PVA) and carboxymethyl cellulose (CMC). They are examples of biopolymers used to produce active films, and both have been investigated for use in active packaging individually.^3,15^

Carboxymethylcellulose (CMC) is a biopolymer derived from cellulose,^16,17^ with good film-forming capacity, low cost, and biodegradability. However, it has limitations such as fragility and high water solubility.^18^ Poly­(vinyl alcohol) (PVA), on the other hand, is a synthetic, semicrystalline, water-soluble, nontoxic, biodegradable polymer that is more expensive than CMC. Its films stand out for their good tensile strength, flexibility, transparency, thermal stability, and good oxygen barrier properties, despite their water solubility.^3^ However, studies indicate that the synergy between the characteristics of PVA (strength, flexibility, and biodegradability) and CMC (absorption, adhesion, and stability) can result in a final polymer with optimized properties for various industrial uses.

Addition of SiO_2_ nanoparticles to PVA/CMC films^19^ increases the refractive index and surface roughness and slightly alters the optical properties. Films produced by incorporation of *Glycyrrhiza glabra* essential oil^20^ reduces mechanical strength but increases antibacterial activity, in addition to improving water vapor permeability. Authors^21^ investigated CMC/PVA membranes demonstrated that the blend promoted a better balance between mechanical strength and flexibility, in addition to reducing water solubility compared to pure films, highlighting the complementarity between the two polymers. These studies showed the potential of combining these two polymers to obtain polymer films with different characteristics when compared to pure polymer films.

Studies have also shown that PVA/CMC blends enriched with bioactive extracts exhibit significant improvements in the physical properties of the films, as well as potential antioxidant and antibacterial benefits that enable their use as food packaging. Kanatt and Makwana (2020)^22^ developed CMC/PVA films incorporated with aloe vera and found that the presence of the extract conferred greater water resistance, as well as antioxidant and antimicrobial properties, suggesting potential for extending the shelf life of foods. Alshehri et al. (2024)^23^ investigated the combination of PVA and CMC fortified with broccoli sprout seeds, and the films offered improved mechanical strength and moisture barrier properties. These films also demonstrated antioxidant and antibacterial effects. This CMC/PVA and active compound association justifies the relevance of the present study, which combines the potential of the polymer matrix with the functionality of sodium nitrite (NaNO_2_). The incorporation of sodium nitrite (NaNO_2_) gives the system an active function, associated with color preservation and reduced lipid oxidation in meats. Thus, the PVA/CMC/NaNO_2_ blend presents itself as a promising strategy for the development of active films applicable to food preservation.

Previous studies^2,24,25^ have shown that NaNO_2_ has a significant influence on the properties of polymeric films; however, its effect on the properties of CMC/PVA blends has not yet been studied. It should be noted that each polymer base has different properties, and factors such as the combination of blends and the addition of additives significantly influence the properties of polymers.^26^ When it comes to active packaging, the polymer matrix is primarily responsible for the release of the active compound. Therefore, studies are needed to elucidate the properties of this new material and assess its applicability. Thus, this study aims to characterize the physical properties of PVA/CMC blends containing different concentrations of NaNO_2_ and investigate their applicability as an active film.

## 2 Materials and Methods

### 2.1 Preparation of the Blends

The CMC/PVA blends were produced using the casting technique.^3^ Initially, the PVA (Dinâmica LTDA, Brazil) was dispersed in distilled water at a concentration of 10% (w/v) with stirring at 200 rpm and a temperature of 90 ± 5 °C for 90 min. The CMC dispersion was prepared at a concentration of 1% (m/v) under stirring at 200 rpm and a temperature of 90 ± 5 °C for 1 min. After preparation, the dispersions were mixed in a 50%/50% ratio, and concentrations of NaNO_2_ 0, 50, 100, and 500 ppm (w/v) and 5% glycerol were added under constant stirring at 200 rpm for 1 min. After preparing the dispersions, the mixtures were placed on glass plates at room temperature (25 ± 5 °C) for 7 days without control the relative humidity of the location, for solvent evaporation and film formation. Three replicates were made for each mixture. After drying, the films were removed from the plates and stored in a desiccator containing barium chloride at a relative humidity close to 0% until their subsequent characterization.^27^

### 2.2 Film Characterization

#### 2.2.1 Thickness

The thickness of the films was assessed using a digital micrometer (Pantec, Model IP54, São Paulo, Brazil) with a resolution of 0.001 mm. The measurements were made by averaging the thickness of 8 different points on the films. The values were expressed in millimeters (mm).

#### 2.2.2 Water Vapor Permeability

The water vapor permeability of the films was determined based on ASTM E96–00,^28^ with modifications. The films were cut into 4 × 4 cm^2^ and placed in containers with a diameter of 2.5 cm containing silica gel inside. The containers were weighed and placed in a desiccator containing distilled water to obtain a relative humidity of approximately 100% at a temperature of 22 ± 3 °C. The containers were weighed every 24 h until a constant weight was reached (17 days). Water vapor permeability (P) was calculated using eq 1.

1 $$P\left(g/m\cdot d\cdot Pa\right)=\frac{\left(r/A\right)\times E}{p\times \left(R_{1}-R_{2}\right)}$$

where *r* is the slope of the regression line (g/d), *A* is the permeation area (m^2^), *E* is the film thickness (m), *p* is the water saturation vapor pressure, *R*_1_ is the relative humidity inside the desiccators, and *R*_2_ is the relative humidity inside the jars.

#### 2.2.3 Water Solubility

To determine solubility, the films were cut to 2 × 2 cm^2^ and dried at 105 ± 5 °C for 24 h. Afterward, the films were weighed, and the mass was recorded (*M*_i_). They were then placed in 50 mL of distilled water at room temperature (25 ± 5 °C) for 24 h. After this time, the films were dried again in an oven at 105 °C for 24 h, and the mass was weighed and recorded (*M*_f_).^29^ The solubility of the films was calculated using eq 2

2 $$solubility\left(\%\right)=\frac{M_{i}-M_{f}}{M_{i}}\times 100\%$$

#### 2.2.4 Color Analysis

To evaluate the color of the films, a Color Quest XE Colorimeter (Hunter Lab, USA) was used in reflectance mode, illuminant D65, 10° observer angle, and 25 cm aperture.^30^ The differences in total color (Δ*E*) were calculated according to eq 3

3 $$\Delta E=\sqrt{{\left(L_{0}-L^{*}\right)}^{2}+{\left(a_{0}-a^{*}\right)}^{2}+{\left(b_{0}-b^{*}\right)}^{2}}$$

where *L*_0_, *a*_0_, and *b*_0_ correspond to the color coordinates for the films without NaNO_2_, and L*, a*, and b* are the color coordinates for the movie with NaNO_2_.

#### 2.2.5 Opacity

The transmittances of the films were measured using a spectrophotometer (Model PC, Shimadzu, Kyoto, Japan) set to a wavelength of 670 nm.^31^ Opacity was calculated using eq 4

4 $$opacity=T_{670}/r$$

where *T*_670_ is the transmittance at a wavelength of 670 nm, and *r* is the film thickness.

#### 2.2.6 Mechanical Properties

The mechanical properties were determined using Brookfield equipment (Model CT3, USA) with a maximum load of 25 kg in accordance with ASTM D882–12.^32^ The films were cut into 25 × 100 mm sizes. The clamp separation was 50 mm, and the deformation speed was 0.4 mm/s. Tensile strength and elongation values were obtained based on eqs 5 and 6, respectively

5 tensilestrength=F/A

6 elongation=A/C

where *A* is the cross-sectional area of the film (width × thickness) (*m*_2_), *F* is the maximum force recorded (*N*), and *C* is the initial length of the film (*m*). The modulus of elasticity was calculated from the slope of the initial portion of the stress–strain curve, which corresponds to the stress divided by the deformation of the film sample.

#### 2.2.7 Fourier Transform Infrared Spectroscopy

FTIR analyses have been modified.^33^ The spectra were obtained in absorbance mode using a Cary630 FTIR spectrometer (Agilent Technologies Inc., Santa Clara, CA, USA), reading in the 4000–400 cm^–1^ region with a resolution of 4 cm^–1^ at room temperature (25 ± 5 °C).

#### 2.2.8 Thermal Analysis

Thermogravimetric analysis (TCA) and differential scanning calorimetry (DSC) were used to characterize the blends thermally. The equipment used for both studies was the LINSEIS TGA/DSC Test Instrument calorimeter (Model STA PT—1000, Simultaneous, Germany). Twenty mg of the film was used, and the temperature range used was 32 to 800 °C with a heating rate of 10 °C/min in an air atmosphere.

### 2.3 Film Application on Pork

To assess the active function of the films, they were applied to pork. Pork loin (*Longissimus lumborum*) purchased from a local supermarket in the city of Itapetinga, Bahia, Brazil, was used. The loin pieces were transported in a thermally insulated box to the laboratory, and the experiments were carried out on the same day.

The pork loins were cut to a thickness of approximately 2 cm and placed in pairs on polystyrene trays, where they were coated with films containing different concentrations of NaNO_2_ (0, 50, 100, and 500 ppm). All the trays were covered with commercial polyvinyl chloride (PVC) film. One treatment also consisted of pieces of loin placed in the trays and coated only with PVC (Positive Control). The samples were stored under refrigeration at 4 ± 2 °C and analyzed on days 0, 3, and 6. Each treatment was analyzed in triplicate with duplicate measurements (*n* = 6 per treatment and day).

#### 2.3.1 Residual Nitrite

Samples of 5 g of pork were ground with 40 mL of distilled water and heated in a water bath at 80 °C for 2 h. After cooling to room temperature, the suspension was filtered through filter paper (UNIFIL, 3 μm pore size) and diluted in a 250 mL volumetric flask. Then, 2 mL were filtered and mixed with 1 mL of distilled water and 0.2 mL of 1% sulfanilamide solution (w/v 0.5 M hydrochloric acid). The tubes were kept in the dark for 5 min, and then 0.2 mL of N-(1-naphthyl)-ethylenediamine (NED) (0.5% w/v) was added, and the mixture was kept in the dark for 15 min. After this time, the mixture was read in a spectrophotometer (Model PC, Shimadzu, Kyoto, Japan) at a wavelength of 540 nm.^34^ The residual nitrite values were calculated based on the NaNO_2_ analytical curve expressed in mg of NaNO_2_/kg of meat.^35^ (*y* = 2.48 × 10^4^ + 0.14*x*; *R* ^2^ = 0.997).

The samples that were packaged only with PVC film (positive control) and with PVA/CMC 0 film were not evaluated for residual nitrite due to the absence of nitrite in the composition of these samples. As a result, no residual nitrite analysis was performed, since this evaluation is only relevant for samples containing nitrite. Residual nitrite analysis was performed only on samples with nitrite additives (50, 100, and 500 ppm), which had measurable nitrite concentrations for this analysis.

#### 2.3.2 Lipid Oxidation

Lipid oxidation was determined according to the methodology using the thiobarbituric acid reactive substance (TBARS) method.^36^ TBARS solutions were prepared at a concentration of 0.08 mol/L dissolved in 50% acetic acid. For the analysis, 10 g of meat was weighed and ground in 40 mL of 5% (w/v) trichloroacetic acid (TCA) and 1 mL of 0.15% (w/v ethanol) BHT. The mixture was filtered through filter paper (UNIFIL, 3 μm pore size) and added to a 50 mL volumetric flask. The volume was topped up with 5% (w/v) TCA. An aliquot of 2 mL was removed and added to a test tube along with 2 mL of 0.08 mol/L thiobarbituric acid. The mixture was homogenized by manual stirring in a water bath at 100 °C for 5 min. The mixture was then cooled to room temperature, and the absorbance was read at 532 nm using a spectrophotometer (Model PC, Shimadzu, Kyoto, Japan). The malondialdehyde (MDA) content was quantified according^37^ to eq 7

7 $$TBARS=\frac{A_{532}\times V\left(L\right)\times 10^{-6}\left(\mu \;mol\;de\;MDA\right)}{1.56\times 10^{5}\left(L/mol\;of\;MDA\right)\times m\left(gmeat\right)\times 1\left(mol\;of\;MDA\right)}$$

where *A*_532_ is the absorbance value at a wavelength of 532 nm, *V* is the volume (*L*), *m* is the mass of the meat, and 1.56 × 10^5^ M/cm is the MDA molar extinction coefficient. The results were expressed in μmol of meat MDA/g.

#### 2.3.3 Meat Color

Meat color was determined by digital image analysis.^38^ Images of the samples were taken using a Samsung Galaxy J5 smartphone in a closed room with only fluorescent lamps for lighting. The distance between the camera and the samples was approximately 15 cm. The images were analyzed using Color Grab software version 3.9.2 (2012). The measurements were based on CIELab coordinates, and the color measurements were determined by averaging five different points on the samples. Saturation (C*) and hue angle (h) were calculated using eqs 8 and 9.

8 $$C^{*}=\sqrt{a^{*2}+b^{*2}}$$

9 $$h=\arctan\frac{b^{*}}{a^{*}}$$

### 2.4 Statistical Analysis

The data from the blends were evaluated in a completely randomized design, analyzed by analysis of variance (ANOVA) and the Tukey mean comparison test, considering a significance level of 5%. In the study of residual nitrite and lipid oxidation, the results were evaluated by descriptive analysis, with at least three replicates. The color analysis of the packaged meats was carried out in a completely randomized design, in a 5 (treatments) × 3 (days) factorial scheme, with three replications. When significant (*p* < 0.05), the means were separated using the Duncan test. Pearson’s correlation coefficients were calculated for the correlation analysis between the residual nitrite, TBARS, a*, C, and h parameters. The significance of the correlation coefficients was tested using the *t*-test for correlation, *p* ≤ 0.05, *n* = 60.

## 3 Results and Discussion

### 3.1 Film Properties

Table 1 shows the physical properties of (PVA/CMC) blends with different concentrations of NaNO_2_. The results show the effect of varying nitrite concentrations in the films compared to the control film (without nitrite addition). ANOVA evaluated all the results with a significance level of 0.05. Among the results, the presence of nitrite significantly influenced the b* value. The film containing nitrite showed a greater tendency to yellow than the film without nitrite (PVA/CMC 0). For the other assessments, there was no variation in characteristics.

**Table 1:** Thickness, Water Vapor Permeability (*P*), Color (L*, a*, b*, Δ*E*, WI), Opacity, Mechanical Properties of Poly­(vinyl alcohol)/Carboxymethyl Cellulose (PVA/CMC) Films Incorporated with NaNO_2_ at Concentrations of 0, 50, 100, and 500 ppm

proprieties	PVA/CMC 0	PVA/CMC 50	PVA/CMC 100	PVA/CMC 500
thickness (mm)	0.12 ± 0.01^a^	0.12 ± 0.01^a^	0.12 ± 0.01^a^	0.12 ± 0.01^a^
*P* × 10^–5^ (g/m·d·Pa)	3.20 ± 0.36^a^	3.22 ± 0.26^a^	3.23 ± 0.37^a^	4.05 ± 0.31^a^
solubility (%)	100	100	100	100
L*	92.28 ± 0.16^a^	92.06 ± 0.04^a^	92.19 ± 0.06^a^	92.28 ± 0.22^a^
a*	–0.89 ± 0.02^a^	–0.89 ± 0.02^a^	–0.93 ± 0.04^a^	–0.96 ± 0.04^a^
b*^a^	1.26 ± 0.04^b^	1.54 ± 0.06^a^	1.55 ± 0.11^a^	1.58 ± 0.15^a^
Δ*E*	1.23 ± 0.13^a^	1.50 ± 0.03^a^	1.48 ± 0.24^a^	1.53 ± 0.17^a^
WI	92.13 ± 0.15^a^	91.62 ± 0.42^a^	91.99 ± 0.08^a^	92.06 ± 0.25^a^
RT (MPa)	28.83 ± 5.65^a^	25.66 ± 3.23^a^	27.62 ± 1.61^a^	27.37 ± 0.25^a^
elongation (%)	179.50 ± 12.00^a^	178.55 ± 2.90^a^	179.03 ± 5.41^a^	179.58 ± 10.22^a^
ME (MPa)	38.92 ± 1.72^a^	37.20 ± 4.85^a^	43.01 ± 5.47^a^	44.19 ± 1.53^a^

a Indicate differences between the lines (*p* < 0.05); water vapor permeability (*P*); L* (luminosity), a* (relative green to red), b* (relative yellow to blue). Total color difference (△*E*) and whiteness (WI). Tensile strength (RT); Modulus of elasticity (ME).

The thickness of PVA/CMC films with different nitrite concentrations remained constant at 0.12 ± 0.01 mm for all samples. This indicates that the addition of nitrite did not affect the thickness of the films, maintaining uniformity. All the films showed total solubility in water after 8 h of exposure. Previous studies^39,40^ demonstrated that both PVA and CMC films are water-soluble due to the presence of hydrophilic groups (hydroxyl) capable of forming hydrogen bonds with water molecules, and the mixture retained this characteristic. The solubility of the blended films in water can be advantageous for promoting faster migration of the active compound into the food. In addition, solubility contributes to the biodegradability characteristics of the polymer.^16^ In the present study, Δ*E* values ranged from 1.23 to 1.53, so there was no color variation between the films that was perceptible to the naked eye. Δ*E* value > 7 indicates a color variation perceptible to the human eye; therefore, for the results presented, the color changes of the blends are not perceptible to the human eye.^41^

The films developed presented water vapor permeability (WVP) values in the range of 3.20 × 10^–5^ to 4.05 × 10^–5^ *P* (g/m·d·Pa), which demonstrates that the films developed have high water vapor permeability under the conditions studied. The water vapor permeability of PVA and CMC films depends heavily on the formulation, relative humidity, and the presence of plasticizers or additives, making it essential to control these variables for specific applications in packaging or active films. The intermolecular interactions, such as hydrogen bonds between PVA and CMC chains, reduce free space and intermolecular distances in the film matrix, influencing the diffusion of water molecules more and WVP.^42^ The experiment was conducted under conditions of 100% relative humidity. Films subjected to lower relative humidity (75%) would generally be more stable and have lower WVP values. In this study, the polymer concentrations were the same. Other researchers have observed that in PVA/CMC blends, higher concentrations of PVA, relative to the amount of CMC, produce films with lower WVP. This is attributed to a more uniform and pore-free film matrix and fewer available hydroxyl groups for moisture passage, resulting in higher hydrophobicity and less water vapor transmission.^43^ Comparing the control film to the films with added nitrite, it can be observed that there were no differences between the WVP values, indicating that the additive did not interact physically or chemically with the blend, modifying neither the structure nor the interaction of the polymer matrix, thus not altering the passage of water vapor through these films. It is therefore observed that PVA/CMC films can be engineered to have favorable WVP by adjusting polymer proportions and using cross-linkers, with higher CMC and adequate cross-linking generally reducing water vapor permeability.

It can be observed that the addition of NaNO_2_ did not alter the mechanical properties of the films obtained by blending (*p* > 0.05) when compared to the film without nitrite. The tensile strength and elongation values of the films show that their structural integrity and flexibility were maintained, and there was no increase in brittleness, i.e., the films did not become more susceptible to breakage under mechanical stress due to the addition of nitrite. The modulus of elasticity also remained unchanged at different nitrite concentrations. PVA/CMC films are formed through hydrogen bonds between the hydroxyl groups of PVA and the hydroxyl and carboxyl groups of CMC, resulting in a stable and homogeneous polymer network.^44^ When incorporated, NaNO_2_ may not disrupt the existing hydrogen interactions between PVA and CMC. Thus, the polymer network structure is not significantly modified, which explains the absence of relevant changes in the mechanical properties observed. High elastic modulus values indicate fragile films with lower elongation,^45^ which was not the case with the PVA/CMC blends in this study.

In previous studies, films developed using only PVA exhibited tensile strength of 35 MPa, elongation of 205.5%, and elastic modulus of 400 MPa. These mechanical properties are superior to those of films obtained with CMC alone produced using traditional casting techniques (tensile strength of 15.8 MPa and elongation of 11.62%).^16^ In the present study, the PVA/CMC combination resulted in films with characteristics closer to those of PVA films, giving the blends strength and flexibility, even after the addition of NaNO_2_. This mixture proved to be adequate, resulting in a film with better mechanical properties when compared to separate polymer bases, especially CMC. Although other techniques such as the flow casting method can optimize the process and also produce cellulose-derived films with better mechanical properties.^46–48^ The improvement in the mechanical properties of films produced with sustainable polymer bases meets the demand for materials that resist storage and transportation and can be used commercially to replace synthetic polymers.

The results of the FTIR analysis of the blends are shown in Figure 1a and Table 2. The peaks at 3282 cm^–1^ and 2939 cm^–1^ are attributed to the stretching vibration of the free O–H and C–H group, respectively, of the CMC film and PVA.^40,49^ The peak at 1595 cm^–1^ refers to the COO band of CMC.^50^ The peaks at 1046 and 1023 cm^–1^ are attributed to C–O–C stretching vibrations, characteristic of the chemical structure of CMC.^51^ The characteristic peaks of PVA are attributed to the vibrations at 1734 and 1718 cm^–1^ of the carbonyl group (C=O) of the acetate groups, which are residues of the hydrolysis of poly­(vinyl acetate) in the manufacture of PVA.^52^ Peaks at 1420 and 1325 cm^–1^ confirm the HC–CH_2_ and CH_2_ stretching vibration of the PVA structure.^53^ At 1088 cm^–1^ is the peak of the C–O stretching vibration of PVA. The peaks at 946 cm^–1^ and 834 cm^–1^ are attributed to the C–O–C group of glycerol.^52^ No changes or new peaks were observed after adding NaNO_2_. This suggests that sodium nitrite did not establish covalent interactions with the PVA/CMC polymer matrix, being physically dispersed in the polymer spaces, a result consistent with previous studies.^3,54^ However, it is important to note that the absence of characteristic O–N=O peaks in the spectra may also be related to the relatively low nitrite concentrations used (≤500 ppm), which may be below the FTIR detection limit.

**Figure 1:**
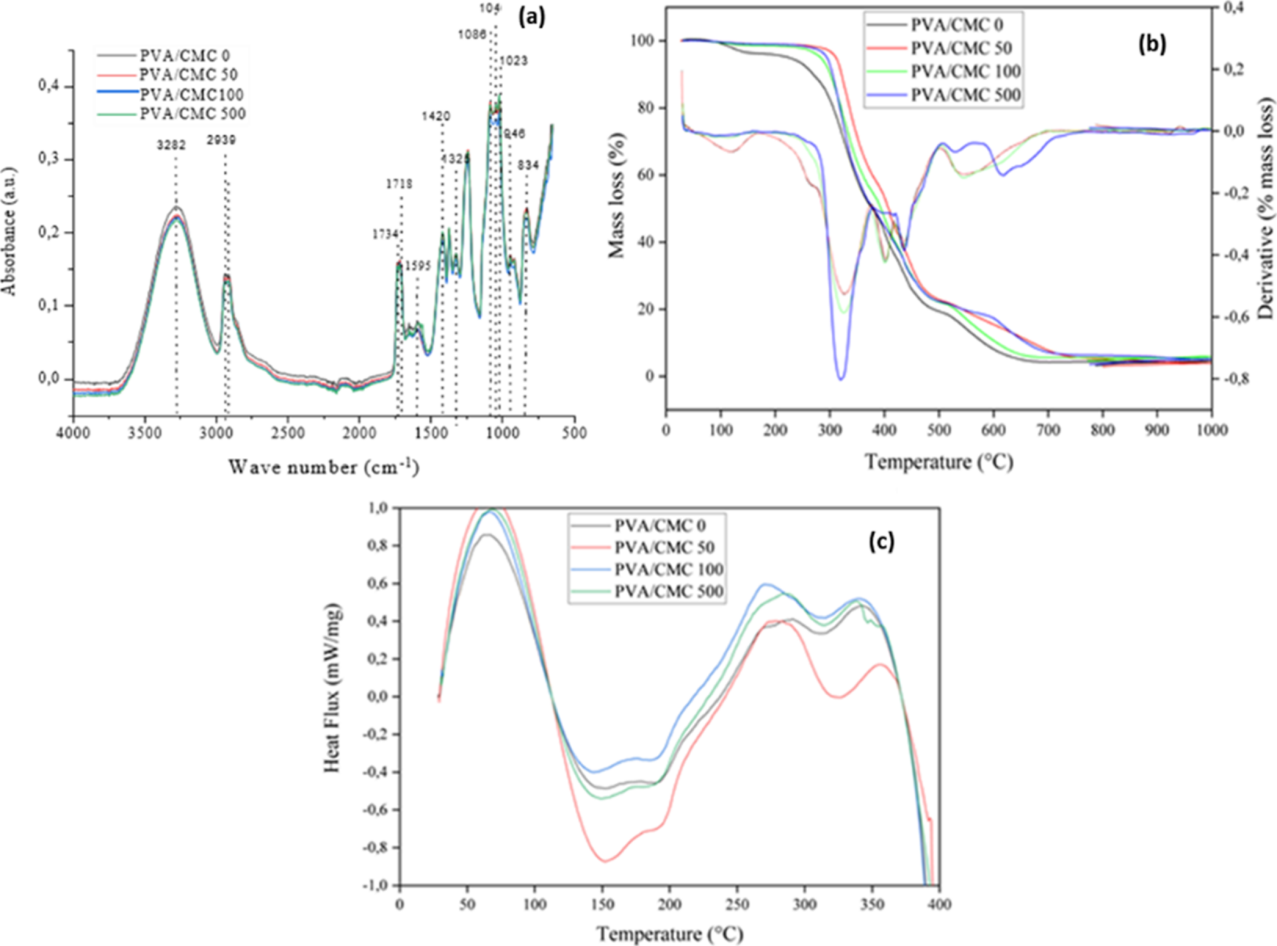
Fourier transform infrared spectroscopy (FTIR) (a), thermogravimetric (b) and differential scanning calorimetric (c) analysis of poly­(vinyl alcohol)/carboxymethyl cellulose blends with 0, 50, 100, and 500 ppm sodium nitrite (NaNO_2_).

**Table 2:** FTIR Bands of Poly­(vinyl alcohol)/Carboxymethyl Cellulose (PVA/CMC) Films Incorporated with NaNO_2_ at Concentrations of 0, 50, 100, and 500 ppm

peak (cm^–1^)	assignment	group	reference
3282	O–H stretching vibration	CMC/PVA	^37,40^
2939	free C–H stretching vibration	CMC/PVA	^37,40^
1595	COO band	CMC	^41^
1046, 1023	C–O–C stretching vibration	CMC	^42^
1734, 1718	vibration of the carbonyl group (C=O) of the acetate groups	PVA	^18^
1420, 1325	HC–CH_2_ and CH_2_ stretching	PVA	^43^
1088	C–O stretching vibration	PVA	^18^
946, 834	C–O–C vibration	glycerol	^18^

The thermal analyses, thermogravimetry (TGA), and differential scanning calorimetry (DSC) are shown in Figure 1b. A loss of mass is observed in the TGA analysis with a peak at 112 °C for the nitrite-free blend (PVA/CMC 0), which is associated with the evaporation of water.^55^ The nitrite-containing blends remained stable over the water evaporation temperature range (70–170 °C), suggesting that water loss occurred slowly in the nitrite-containing films. A second mass loss peak was observed in all samples between temperatures 193–374 °C. This second peak is associated with the evaporation of bound water and the degradation of glycerol and CMC.^15^ A third peak was observed in the temperature range 398–454 °C related to the degradation of PVA residues. A final loss of mass occurred between temperatures 505–611 °C, and this loss is associated with the decomposition of the PVA main chain.^55^ Based on the TGA results, it can be seen that the blends containing NaNO_2_ have greater thermal stability, especially in the temperature range of 30–250 °C. This can be attributed to the presence of nitrite in the film spaces, given its high degradation temperature. Furthermore, studies revealed that nitrite can also act as an antioxidant and inhibit polymers’ oxidative degradation by eliminating the free radicals generated during the thermal decomposition process.^56^

Figure 1c shows two endothermic peaks. The first peak at 147 °C is associated with the evaporation of water molecules.^57^ The second peak occurs at 193 °C and is formed due to the melting of the PVA/CMC blend.^16,58^ Exothermic peaks occur at 273 and 357 °C, associated with polymer decomposition processes.^16,17,59^ All processes occurred regardless of the presence of NaNO_2_.

The thermal analysis (DSC/TGA) revealed that all PVA/CMC films exhibited a multistage degradation profile, characterized by an initial mass loss below 120 °C, attributed to moisture evaporation, followed by major decomposition events related to polymer backbone scission. A comparative summary of thermal parameters is presented in Table 3.

**Table 3:** - Comparative Thermal Parameters (*T*_onset_, *T*_max_, and Residual Mass) of PVA/CMC Films

sample (PVA/CMC)	*T*_onset_ (°C)	*T*_max_ (°C)	residual mass (%)	observation
PVA/CMC 0	∼100	∼250/330	∼10–15	two main degradation steps (water loss and polymer backbone decomposition)
PVA/CMC 50	∼110	∼255/335	∼12–14	slight shift to higher *T*_onset_, indicating improved thermal stability
PVA/CMC 100	∼115	∼260/340	∼14–16	higher *T*_max_, suggesting increased resistance to chain scission
PVA/CMC 500	∼120	∼270/350	∼18–20	most thermally stable sample, likely due to stronger intermolecular interactions

As shown, the incorporation of CMC progressively increased the onset degradation temperature (*T*_onset_) and shifted *T*_max_ toward higher values, indicating an enhancement in thermal stability (Table 4). Notably, the PVA/CMC 500 sample exhibited the highest *T*_onset_ (∼120 °C) and *T*_max_ (∼350 °C), suggesting that higher CMC content reinforces polymer interactions and delays chain scission.

**Table 4:** Pearson’s Correlation of Residual Nitrite Properties, TBARS and Colorimetric Coordinates (Lab*)

variable	residual nitrite	TBARS	*a*	*C*	*h*
residual nitrite	3/4				
TBARS	0.282^a^	3/4			
a*	–0.190	–0.175	3/4		
*C*	–0.092	–0.036	0.910^a^	3/4	
*H*	0.262^a^	0.373^a^	–0.643^a^	–0.300^a^	3/4

a *p* < 0.05. Note: the bars represent standard error of the mean; control: polyvinyl chloride (PVC); PVA/CMC: poly­(vinyl alcohol)/carboxymethyl cellulose incorporated with sodium nitrite (NaNO_2_) at concentrations of 0, 50, 100, and 500 mg/kg.

These results are consistent with previous studies reporting that the addition of polysaccharides into PVA matrices enhances hydrogen bonding and restricts polymer chain mobility, leading to improved thermal resistance.^60–62^

### 3.2 Application of Blends in Pork

The results for the evaluation of residual nitrite and lipid oxidation are shown in Figure 2. It can be observed that the residual nitrite (Figure 2a) present in the meat was higher as the concentration of nitrite added to the blend increased. Approximately 60% of the total nitrite had migrated to the meat on the sixth day of analysis. This rapid migration may be associated with the fact that NaNO_2_ was deposited in the free spaces of the polymer matrix, which facilitates diffusion, in addition to the high affinity of the additive with the water present in the meat.^54,63^ Despite this, the residual amounts of nitrite remained below the 150 ppm limit established by international regulatory agencies.^10–12^

**Figure 2:**
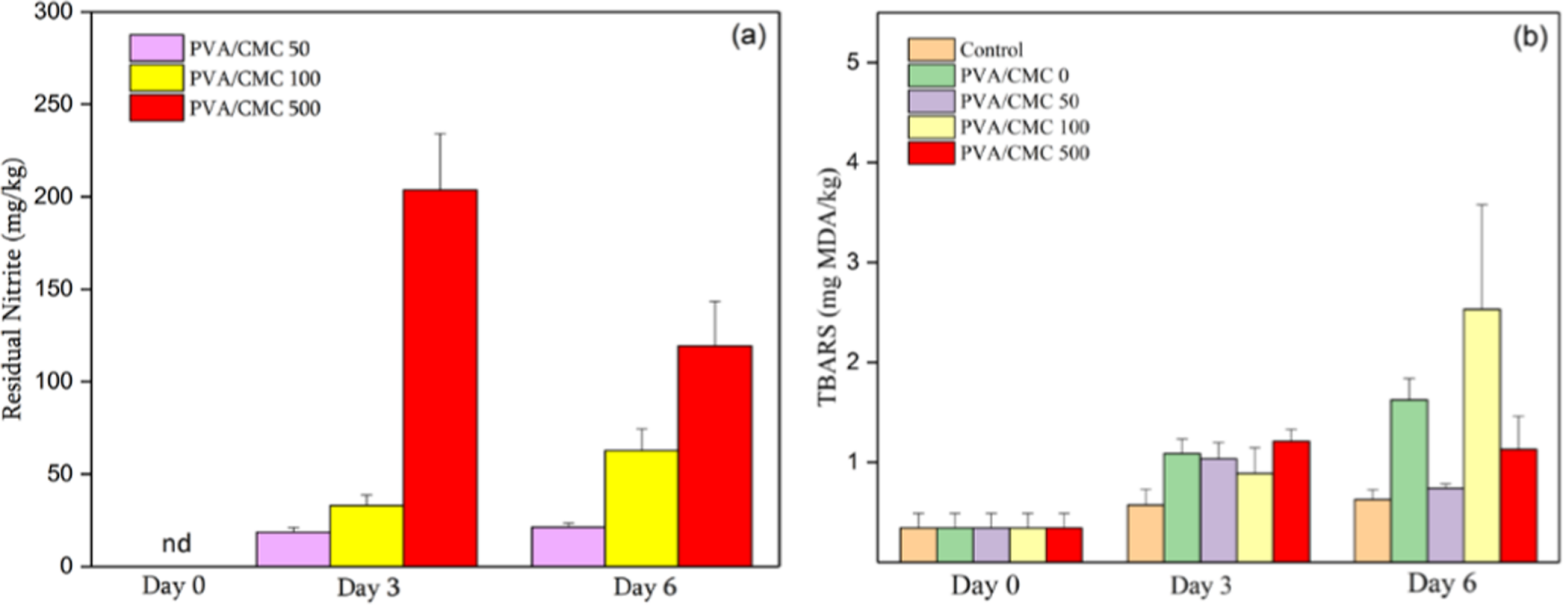
Residual nitrite (a) and TBARS (b) of meat samples packaged with blends of polyvinyl chloride and poly­(vinyl alcohol)/carboxymethylcellulose (PVA/CMC) incorporated with sodium nitrite (NaNO_2_) at concentrations of 0, 50, 100, and 500 ppm.

However, there is an inconsistency in behavior between the 100 and 500 ppm treatments. While the 100 ppm samples showed a reduction on days 3 and 6, the 500 ppm samples showed the opposite trend. This apparently unexpected behavior may be related to variations in the local diffusion of nitrite within the meat, possibly influenced by the structural heterogeneity of the tissue and the distribution of water in the samples, which affects the uneven migration of the additive.^64^

About lipid oxidation, it was observed that, regardless of nitrite concentration, there was no inhibition of peroxidation over time (Figure 2b). This result contradicts previous studies,^2,3^ which demonstrated that nitrite in active packaging systems can reduce lipid oxidation in meat. The solubility of the PVA/CMC blend in water may favor the mobilization of metal ions present in meat (Fe, Cu), thereby promoting the Fenton reaction and the formation of free radicals that accelerate the oxidation of unsaturated fatty acids.^65^ However, it should be emphasized that no direct quantification of these ions was performed in the present study; therefore, this interpretation should be understood as a plausible hypothesis that needs to be confirmed in future investigations. In addition, it should be considered that TBARS values may fluctuate due to methodological interference, such as the reaction of TBA with compounds other than malonaldehyde, or even interaction with meat pigments.^66^

Another possibility is that PVA/CMC-based films exhibited oxygen permeability, which favors diffusion and promotes lipid oxidation in meat. Although oxygen permeability (OTR) analysis was not performed in this study, previous studies have shown that PVA-based systems, when combined with other structuring agents, can significantly reduce oxygen passage.^67–70^ In the present study, the incorporation of NaNO_2_ maintained the structural integrity of the films but did not show any changes that could indicate alterations in OTR.

Recent studies show^71^ that films made of PVA, sodium caseinate, and purified anthocyanin extract from poinsettia leaves applied to ground beef exhibited similar behavior in terms of migration and controlled release, contributing to the preservation of product quality during storage. These findings reinforce the results of the present study, since the PVA/CMC matrix enabled the effective incorporation and migration of nitrite.

The color parameters for the pork samples coated with the active nitrite blends are shown in Figure 3. It was observed that for the parameters studied, a*, *C*, and *h*, no statistical difference was observed on the sixth day of analysis (*p* > 0,05). The lack of statistical difference in the a*, chroma, and hue values indicates that the PVA/CMC blends, regardless of the nitrite concentration, were effective in maintaining the color stability of the meat during storage.^2,3,25,72^ This color stability is particularly relevant for meat, as it is a crucial sensory attribute that significantly influences consumer acceptance.

**Figure 3:**
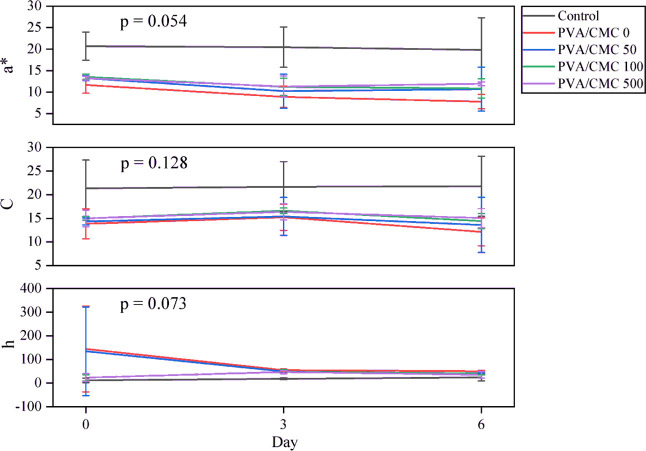
Colorimetric analysis of meat samples coated with PVA/CMC blends containing different concentrations of nitrite and ANOVA analysis for the sixth day of the experiment.

It was visually observed that from the third day of evaluation, the meats packaged with the PVA/CMC blend showed a brownish color, while the meats packaged with the control film did not show this color until the sixth day. This coloration may be associated with the effect of oxidation of the meat’s lipids.^73^ Other researchers have developed active packaging containing NaNO_2_ in polymeric matrices other than PVA/CMC and observed different effects on lipid oxidation and meat color related to the migration of NaNO_2_ from the packaging to the meat.^2,25,72^ However, some of these experiments used vacuum packaging. In this study, the meat samples were packaged in atmospheres with the presence of air. These results show that the type of polymer base, the concentration of nitrite, and the presence of oxygen directly interfered with the quality characteristics of the packaged pork. Compared to the PVA film from the previous study by,^3^ the PVA/CMC blend also promoted a noticeable color change in the meat, showing a darker color with storage. It also promoted an incredible migration of nitrite into the meat under the conditions studied.

Although PVA/CMC films provided good color stabilization, lipid oxidation control was limited, possibly due to their hydrophilic nature and single-layer structure. Future studies may explore the use of multilayer films, polymer matrix cross-linking, or the incorporation of natural antioxidants as strategies to enhance oxidative protection.

These results are not just satisfactory, but they also have significant practical implications. They demonstrate the potential of the PVA/CMC blend in active packaging, which can be applied to various meat products. For instance, it can be used in fresh meat to extend shelf life and display life, or in cured processed products postprocessing.^74^

### 3.3 Correlation Matrix

Table 2 shows the correlation matrix between the properties of meat coated with PVA/CMC blends at different nitrite concentrations. A weak but significant association between residual nitrite and TBARS suggests that increasing residual nitrite increases lipid oxidation. This result contrasts with previous studies claiming that nitrite has antioxidant properties.^3,72^ The positive and significant correlation between residual nitrite and h indicates that higher nitrite concentrations are associated with a higher *h* value, representing the hue angle. This suggests that nitrite can alter the color tone of meat due to the formation of dark-toned compounds from lipid oxidation resulting from the Fenton reaction, a statement confirmed by the presence of a significant correlation between TBARS and *h*, suggesting that lipid oxidation leads to changes in the color tone of meat. The strong correlation between a* and C* confirms that the intensity of a* is closely linked to color saturation. This is to be expected, as both parameters are indicators of the vividness of the color, and the a* value is used mathematically to obtain *C*.^37^ The negative correlation between a* and *h* suggests that an increase in the intensity of the red color is associated with a decrease in the *h* value, indicating a redder and less yellowish hue. This correlation study reinforces the changes in meat characteristics caused by the presence of nitrite.

The correlation between the data showed that although color intensity and saturation are highly correlated with each other, residual nitrite and lipid oxidation play a significant role in altering meat color, which can compromise color stability and, consequently, product acceptance over storage time.

Different combinations of polymer matrices and active compounds have been proposed for application in meat to maintain its quality by preserving color characteristics, preventing oxidation, and also inhibiting the growth of microorganisms.^23,75–78^ In comparison, the film developed in this study, based on PVA/CMC with the addition of NaNO_2_, although it has some barrier properties inferior to conventional plastics and some of these developed films, stands out for its economic viability, as it uses lower-cost and more sustainable raw materials. This PVA/CMC blend can contribute to reducing food waste, since the active compounds incorporated into the polymer matrix preserve food for longer. This means less product disposal throughout the production chain.

Biodegradable films are seen as packaging that will be discarded, rather than packaging to be recycled, unlike nondegradable packaging.^79^ However, when it comes to recycling biodegradable films, challenges such as high costs arise, causing investors to lose interest in this type of packaging.^80^ Nevertheless, technological advances should reduce expenses and alleviate the adverse effects on recyclability, thereby increasing the use of biodegradable polymers. However, the PVA/CMC blends developed, because they are biodegradable due to their solubility and hydrophilic nature, will have a lower environmental impact compared to petroleum-based packaging and may also preserve food.

## 4 Conclusion

The blends showed superior physical properties to the isolated polymers, where the presence of sodium nitrite did not interfere with the physical properties of the blends. When applied to pork, the migration of nitrite into the meat occurred rapidly and mainly altered the color characteristics of the meat. The PVA/CMC blend incorporated with nitrite is suitable for use with fresh meat or in postprocessing cured products. Although the active blends showed promising physical characteristics, lipid oxidation and the influence of the type of film on the meat’s color require further investigation to optimize the active film’s effectiveness and guarantee the pork’s quality during refrigerated storage.
